# Overlooked impact of less severe physical violence on antenatal care visits: Findings from South Asia

**DOI:** 10.7189/jogh.13.04155

**Published:** 2023-11-17

**Authors:** Ling Liu, Di Liang, Saeed Anwar, Zunaira Michael, Shrinkhala Barun Shrestha, Nasrin Sultana, Jiayan Huang

**Affiliations:** 1 School of Public Health, Global Health Institute, Fudan University, Shanghai, China; 2 Prime Institute of Public Health, Peshawar Medical College, Peshawar, Pakistan; 3 School of Medical Sciences, Kathmandu University, Dhulikhel, Nepal; 4 Institute of Health Economics, University of Dhaka, Dhaka, Bangladesh

## Abstract

**Background:**

In South Asia, women often experience intimate partner violence (IPV) and have limited access to maternal health services (MHS). However, the effects of IPV on antenatal care (ANC) visits remain unclear. This study aimed to examine the impact of IPV of different forms and severities on ANC visits in South Asia.

**Methods:**

This cross-sectional study used the latest available data from demographic and health surveys conducted in Bangladesh, India, Afghanistan, Nepal, Maldives, and Pakistan. The study sampled 4467 women who had given birth within the past 12 months and were interviewed for IPV. IPV was measured by binary variables indicating the presence of physical violence (PV), categorised into less severe (LSPV) and severe physical violence (SPV), emotional violence (EV), and sexual violence (SV). ANC utilization was measured using binary variables indicating whether respondents had any, at least four, or at least eight ANC visits, as recommended by World Health Organization (WHO). Logistic regressions adjusted for survey weights were used to assess associations between ANC utilization and exposure to IPV during pregnancy and lifetime.

**Results:**

The prevalence of LSPV, SPV, EV, and SV during pregnancy were 14.5%, 4.4%, 11.6%, and 4.1%. LSPV experience during pregnancy was associated with decreased likelihoods of at least four ANC visits (odds ratio (OR) = 0.55; 95% confidence interval (CI) = 0.40-0.76) and eight ANC visits (OR = 0.53; 95% CI = 0.31-0.90). Results of lifetime exposure to IPV followed similar patterns. Lifetime exposure to LSPV was associated with decreased likelihoods of at least four ANC visits (OR = 0.55; 95% CI = 0.41-0.74) and eight ANC visits (OR = 0.47; 95% CI = 0.29-0.77).

**Conclusion:**

This study highlights the negativities of LSPV on the frequency of women seeking ANC visits. Policies are necessary to identify women at risk of the often-overlooked LSPV early and provide protective interventions to promote maternal health in South Asia.

The underutilization of maternal health services (MHS) in South Asia has significant public health consequences for women and children in this region. Only 31.2% of ever-married women utilized MHS as recommended in Bangladesh [1,2]. The underutilization of antenatal care (ANC) is linked with adverse maternal and child outcomes, such as developing anemia pregnancy and delivering low birth weight babies [3]. As the second highest region in newborn mortality following Sub-Saharan Africa, South Asia accounted for 36% of global newborn deaths with a rate of 23 deaths per 1000 live births [4,5]. In 2020, four of the eight countries in South Asia, namely India, Pakistan, Bangladesh, and Afghanistan, were among the top ten countries with the highest number of newborn deaths [6].

Previous studies have focused on the impact of socioeconomic factors and quality improvement initiatives on MHS utilization, but few have explored the associations between intimate partner violence (IPV) and MHS [7-10]. IPV refers to actions by an intimate partner or ex-partner that cause physical, emotional, or sexual harm, including physical aggressions, controlling behaviors, psychological abuse, and sexual coercion [11]. Exposure to IPV during pregnancy was associated with increased risks of adverse birth outcomes [4,6,12-17]. However, the mechanisms underlying such effects were less understood due to a lack of direct data on exposure to IPV during pregnancy. We proposed that the impacts of during-pregnancy IPV on ANC visits might be one possible explanation. Previous studies examining the relationship between IPV and ANC visits were often localized and may not be generalisable to the whole region [18-22]. Additionally, it was also unclear how the severity of physical violence affects ANC visits, as some studies did not distinguish between severities while others focused solely on severe physical violence [18,20,22-26].

This study aimed to explore the impacts of IPV on the frequency of ANC visits among pregnant women. Moreover, we attempted to distinguish between different timeframes of IPV exposure as well as severities of physical violence to examine the extent of their effects on ANC visits. We hypothesized that all forms of IPV had negative associations with women’s ANC utilization.

## METHODS

### Study design and data sources

This is a cross-sectional study using the demographic and health surveys (DHS). South Asian countries were selected based on United Nations Children’s Fund (UNICEF) official working sites in South Asia, as well as the availability of DHS data sets. All the latest available countries’ DHS and domestic violence data were included, specifically Bangladesh (2007), India (2019-2021), Afghanistan (2015), Nepal (2016), Maldives (2016-2017), and Pakistan (2017-2018) [27].

DHS surveys are nationally representative cross-sectional surveys that collect information from samples of the populations. Surveys employ a random sampling method based on a stratified two-stage cluster design with subgroups (strata) of geographic or administrative subnational regions. Primary sampling units (PSUs) form the survey cluster within each stratum. PSUs are selected with probability proportional to the sample size and consist of census enumeration areas, generally neighborhoods in urban areas and villages in rural areas. Each sample has a sampling weight, defined as the inverse probability of being included in the survey, to adjust for different possibilities of selection or interview, nonresponses, and oversampling due to design or happenstance [28]. Regarding questionnaires used in different countries, DHS policy employed a complicated translation process to ensure validity and reliability of questionnaires in major local languages [29].

In this study we analyzed data sets from the official DHS program website. Those data sets are publicly available and properly anonymised. Informed consent was obtained at the time of original data collection by DHS. The analysis did not involve any secondary use of data. No individuals or biological samples were contacted for this study. Thus, ethical approval did not apply to this study.

### Study sample

**Figure 1:**
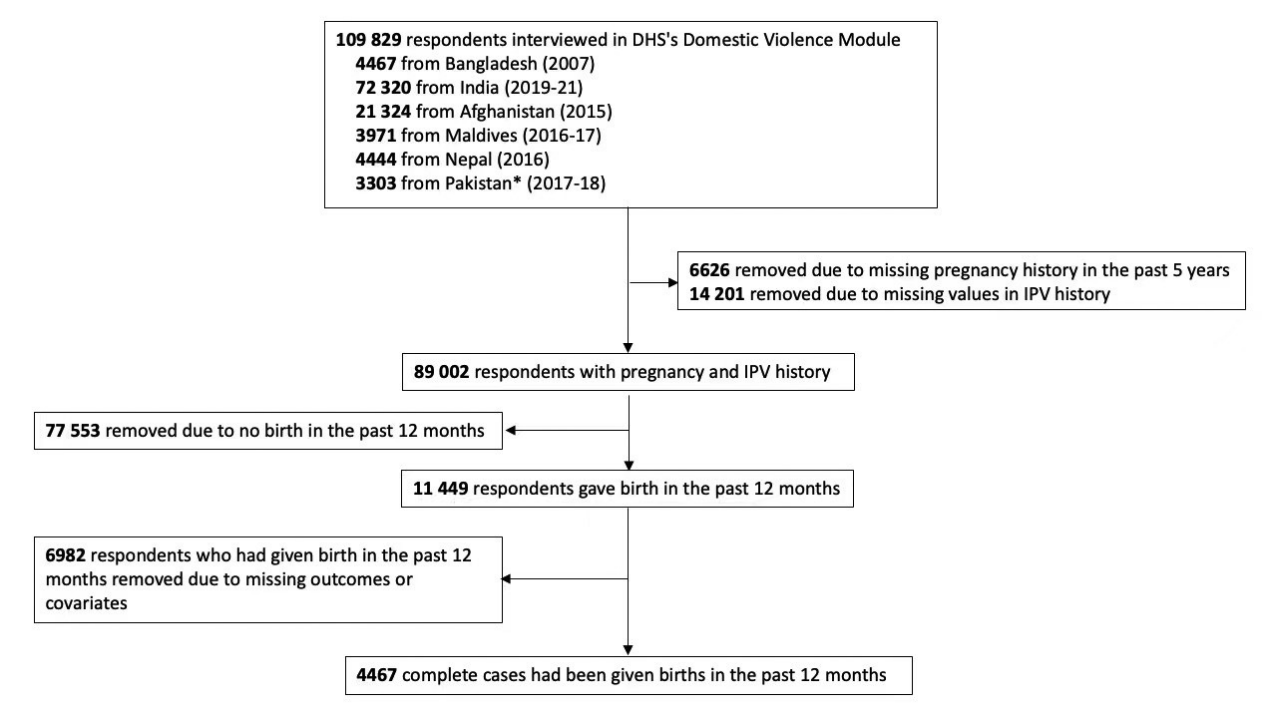
Flowchart of cases selection. Flowchart of cases selection. We excluded 782 records from region Azad Jammu and Kashmir and Gilgit-Baltistan from Pakistan’s data set. DHS – Demographic and health surveys, IPV – intimate partner violence.

The latest available data in South Asia included 109 829 respondents aged 15-49 interviewed for questions on domestic violence. Records missing answers for lifetime exposure to IPV or pregnancy history in the past five years were excluded. In total, 89 002 respondents with IPV and pregnancy history remained in the database. Among those respondents, those who had not given birth in the past 12 months and records missing answers for outcomes or covariates were excluded. Final samples included 4467 complete cases who had given birth in the past 12 months. A flowchart diagram of the selection process is illustrated in **Figure 1**.

### Intimate partner violence

We scrutinised different forms of IPV, including physical violence (PV), emotional violence (EV), and sexual violence (SV). Physical violence (PV) was further categorised into less severe physical violence (LSPV) and severe physical violence (SPV). The presence of LSPV and SPV was determined by whether respondents had experienced any behaviors mentioned in the questionnaires (**Table 1**). Classification of forms of IPV followed the recode manual defined by the DHS program [30]. Answers were coded “1” if “yes” and “0” if “no” to any of the questions. In determining PV, a respondent would be coded “0” if not experienced any LSPV or SPV behaviors, “1” if experienced LSPV only, and “2” if experienced SPV regardless of LSPV.

**Table 1:** Classification of IPV based on partners’ behaviors

Less severe physical violence
Partners’ behavior
*Push, shake or throw something at the respondent*
*Slap the respondent*
*Punch the respondent with a fist or with something that could hurt the respondent*
*Twist the respondent’s arm or pull the respondent’s hair*
**Severe physical violence**
Partners’ behavior
*Kick, drag or beat the respondent up*
*Try to choke or burn the respondent on purpose*
*Threaten or attack the respondent with a knife, gun or any other weapon*
**Emotional violence**
Partners’ behavior
*Say or do something to humiliate the respondent in front of others*
*Threaten the respondent or someone close to the respondent with harm*
*Insult the respondent or make the respondent feel bad about herself*
**Sexual violence**
Partners’ behavior
*Physically force the respondent to have sexual intercourses*
*Physically force the respondent to perform other sexual acts*
*Force the respondent with threats/other way to perform other sexual acts*

We divided timeframes of respondents’ exposure to IPV into during-pregnancy exposure and lifetime exposure. Since we exclusively selected respondents who had been pregnant in the past 12 months, exposure to IPV during pregnancy was a proxy of respondents’ exposure to IPV within the past 12 months.

### Outcomes

Outcome variables in this study were the frequency of ANC visits of respondents’ latest births within the past 12 months. Based on World Health Organization (WHO) focused ANC (FANC) model in 2002 and its updated recommendation in 2016, the frequency of ANC visits of respondents’ latest births was further split into three outcome variables: a) had any ANC visit, b) had at least four ANC visits, 3) had at least eight ANC visits. Variables were coded “1” if the respondent had any ANC visits, had at least four ANC visits, or had at least eight ANC visits, and “0” if not.

### Covariates

According to previous research on possible factors related to IPV and maternal health, we considered covariates from individual and family (household) levels. Individual level covariates included personal information (the respondent’s age, area of residence, education level, working status) and marital and reproduction information (age at first marriage, age at first birth, parity times, marital duration). Family (household) level covariates included wealth index, household decision power, the respondent’s relationship to the household head and the husband’s education level, working status, and desire for children.

To evaluate respondents’ decision power in households, DHS asked whether the respondent had any final say in six decisions – health care, large household purchases, daily needs household purchases, visits to family or relatives, food to be cooked every day, and what to do with money earned by husband. In this study, respondents got one additional score if they had any final say on each of the abovementioned decisions. Respondents would get a minimum score of zero and a maximum score of six. Those scored zero were coded “no decision power”, zero to three were coded “some decision power”, and higher than four were coded “full decision power”.

### Statistical analysis

We applied descriptive analysis to all predictors, covariates, and outcome variables. We used χ^2^ tests to test possible dependency between respondents’ experiences of four forms of IPV and the frequency of ANC visits. In complete cases, we employed logistic regression models adjusted for covariates and survey weights to assess the impacts of four forms of IPV within different exposure timeframes on ANC visits. Specifically, outcomes within the past 12 months were regressed against respondents’ exposure to IPV during pregnancy and respondents’ lifetime exposure to IPV. Assessment of the IPV’s impact significance is based on adjusted odd ratios (OR) with 95% confidence intervals (CIs) calculated by respective regression models. For determining statistical significance, a two-sided *P*-value of 0.05 was set as a cutoff.

We discarded Bangladesh’s data in logistic regression models due to the lack of information on EV and SV. To ensure the robustness and statistical significance of logistic regression models without Bangladesh’s PV data, we further performed sensitivity analyses using Bangladesh’s PV data. Sensitivity analyses regressed PV against each ANC outcome. Additionally, the DHS data collected in India encompassed the coronavirus disease 2019 (COVID-19) pandemic period. We also performed sensitivity analyses discarding India’s PV data to eliminate the potential impact caused by the pandemic. We performed descriptive analysis and implemented logistic regression models using Stata/SE (15.1, StataCorp LLC, College Station, USA). The χ^2^ test was performed in R, version 4.0.5.

## RESULTS

### Sample characteristics at the individual and family levels

There were 4467 respondents who had been pregnant in the past 12 months in South Asia, over 76% lived in rural areas, more than 75% received some education, and the majority were unemployed. Analysis of marital and reproduction information revealed that nearly 90% of respondents married and gave first birth at the age of 15 to 29. Most respondents had borne more than one child (**Table 2**).

**Table 2:** Characteristics of respondents who had given births in the past 12 mo (n = 4467), presented as n (%)

Personal information variables	
Age in years	
*15-19*	252 (5.64)
*20-24*	1638 (36.67)
*25-29*	1545 (34.59)
*30-34*	705 (15.78)
*35-39*	256.73 (5.73)
*40-44*	57 (1.28)
*45-49*	14 (0.31)
Area of residence	
*Urban*	1050 (23.51)
*Rural*	3417 (76.49)
Education level	
*No education*	1090 (24.4)
*Primary*	524 (11.73)
*Secondary*	2096 (46.92)
*Higher*	757 (16.95)
Working status	
*Unemployed*	3707 (82.99)
*Employed*	760 (17.01)
**Marital and reproductive variables**	
Age at first marriage	
*<15*	220 (4.93)
*15-19*	2108 (47.19)
*20-24*	1623 (36.33)
*25-29*	440 (9.85)
*30-34*	64 (1.43)
*35-49*	12 (0.27)
Age at first birth	
<15	30 (0.67)
*15-19*	1284 (28.74)
*20-24*	2208 (49.43)
*25-29*	763 (17.08)
*30-34*	148 (3.31)
*35-49*	34 (0.76)
Parity	
*Uniparous*	1711 (38.3)
*Multiparous*	2756 (61.7)
Marital duration (years)	
*0-4*	2249 (50.35)
*5-9*	1344 (30.09)
*10-14*	567 (12.69)
*15-19*	214 (4.79)
*20-24*	80 (1.79)
*35-29*	13 (0.29)
*30+*	0 (0)
**Family (household) variables**	
Wealth index	
*Poorest*	1141 (25.54)
*Poorer*	1060 (23.73)
*Middle*	840 (18.80)
*Richer*	776 (17.37)
*Richest*	650 (14.55)
Decision power in household	
*No decision power*	*772 (17.28)*
*Some decision power*	*1194 (26.73)*
*Full decision power*	*2501 (55.99)*
Relationship to household head	
*Head*	*267 (5.98)*
*Wife*	*1940 (43.43)*
*Other*	*2260 (50.59)*
*Not related*	0 (0)
Husband’s education level	
*No education*	701 (15.69)
*Primary*	568 (12.72)
*Secondary*	2272 (50.86)
*Higher*	926 (20.73)
Husband’s working status	
*Unemployed*	2242 (50.19)
*Employed*	2225 (49.81)
Husband’s desire for child	
*Same or more*	3736 (83.64)
*Fewer*	731 (16.36)

For respondents’ family structure, 48% were poor (poorest and poorer), about 31% were rich (richer and richest), nearly half were wives of household heads, and more than 55% had full decision power in households. Of respondents’ husbands, nearly 85% attended at least primary school, about 50% were employed, and about 83% wanted the same number of or more children compared to respondents’ desires (**Table 2**).

### Intimate partner violence prevalence and antenatal care visits in South Asia

Among 4467 respondents who had given birth in the past 12 months in South Asia, about 14.5% experienced LSPV during pregnancy, 4.4% experienced SPV during pregnancy, 11.6% experienced EV during pregnancy, and 4.1% experienced SV during pregnancy (**Table 3**). Moreover, most respondents suffered both IPV lifelong and during pregnancy. Less than 4% of respondents were exposed to only lifetime IPV (**Table 4**). In different countries, during pregnancy exposure to LSPV ranged from 3.8% (Maldives) to 27.5% (Afghanistan), SPV ranged from 0.8% (Maldives) to 11.6% (Afghanistan), EV ranged from 6.9% (Maldives) to around 33.9% (Afghanistan), SV ranged from 0% (Maldives) to 8.5% (Afghanistan) (Table S1 in the **Online Supplementary Document**).

**Table 3:** Intimate partner violence status of respondents who had given births in the past 12 mo under different timeframes (n = 4467), presented as n (%)

Exposure to intimate partner violence in different timeframes	During pregnancy	Lifetime exposure
Physical violence		
*No*	3623 (81.11)	3449 (77.21)
*Less severe physical violence*	646 (14.46)	776 (17.37)
*Severe physical violence*	198 (4.43)	242 (5.42)
Emotional violence		
*No*	3951 (88.45)	3878 (86.81)
*Yes*	516 (11.55)	598 (13.39)
Sexual violence		
*No*	4282 (95.86)	4262 (95.41)
*Yes*	185 (4.14)	205 (4.59)

**Table 4:** Intimate partner violence status of respondents who had given births in the past 12 mo under different timeframes (n = 4467), presented as n (%)

Timeframes of respondents being exposed to intimate physical violence	
Physical violence	
*No exposure*	3466 (77.59)
*Only lifetime less severe physical violence exposure*	147 (3.29)
*Both during pregnancy or lifetime less severe physical violence exposure*	629 (14.08)
*Only lifetime serious physical violence exposure*	27 (0.60)
*Both during pregnancy or lifetime serious physical violence exposure*	198 (4.43)
Emotional violence	
*No exposure*	3878 (86.81)
*Only lifetime exposure*	73 (1.63)
*Both during pregnancy or lifetime exposure*	516 (11.55)
Sexual violence	
*No exposure*	4262 (95.41)
*Only lifetime exposure*	20 (0.45)
*Both during pregnancy or lifetime exposure*	185 (4.14)
At least one type of intimate partner violence	
*No exposure*	3323 (74.39)
*Only lifetime exposure*	174 (3.90)
*Both during pregnancy or lifetime exposure*	970 (21.71)

**Figure 2:**
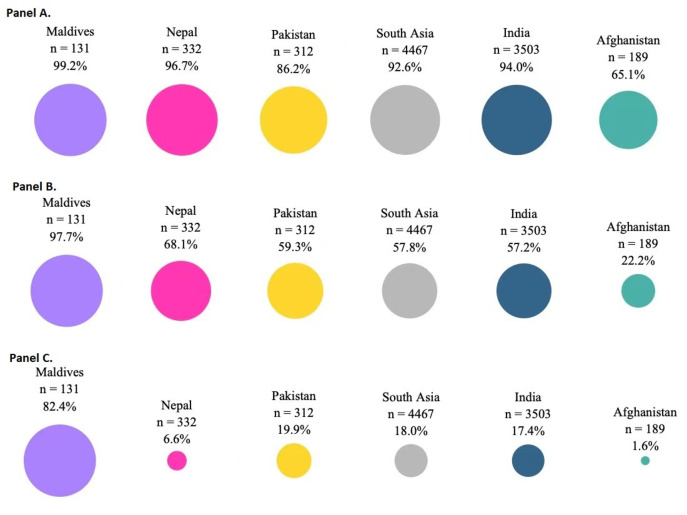
Antenatal care visits in the past 12 months by country. **Panel A**. Percentage of respondents who had at least one antenatal care visit (>0). **Panel B**. Percentage of respondents who had at least four antenatal care visits (≥4). **Panel C**. Percentage of respondents who had at least eight antenatal care visits (≥8).

Regarding conditions of ANC visits, of 4467 respondents who had given birth in the past 12 months, 92.6% had some ANC visits, 57.8% visited ANC at least four times, 18% visited ANC at least eight times. Percentages range greatly in different countries. Afghanistan had the worst and Maldives had the best ANC visit compliance (**Figure 2**). Less ANC visits were observed among respondents who experienced some form of IPV in their lives and during pregnancy in the past 12 months. Of respondents who experienced some form of IPV in their lives, about 90% had ANC visits, 45% visited ANC at least four times, 10% visited ANC at least eight times. While these percentages were over 92%, 58%, and 18% for respondents who did not experience a specific form of IPV, suggesting higher ANC utilization (**Table 5**).

**Table 5:** Distribution of antenatal care visits by intimate partner violence status for respondents who gave birth in the past 12 months (n = 4467), presented as n (%)

ANC visits in the past 12 months	LSPV during pregnancy (n = 646)	SPV during pregnancy (n = 198)	EV during pregnancy (n = 516)	SV during pregnancy (n = 185)	No PV during pregnancy (n = 3623)*	No EV during pregnancy (n = 3951)	No SV during pregnancy (n = 4282)
Had any ANC visits (>0) for the latest birth	587 (90.9)	184 (92.9)	461 (89.3)	164 (88.6)	3366 (92.9)	3676 (93)	3973 (92.8)
Had at least four ANC visits (≥4) for the latest birth	282 (43.7)	96 (48.5)	229 (44.4)	85 (45.9)	2206 (60.9)	2355 (59.6)	2499 (58.4)
Had at least eight ANC visits (≥8) for the latest birth	62 (9.6)	17 (8.6)	56 (10.9)	18 (9.7)	726 (20)	749 (19)	787 (18.4)

ANC – antenatal care, EV – emotional violence, LSPV – less severe physical violence, PV – physical violence, SPV – severe physical violence, SV – sexual violence

*Respondents did not experience either less severe or severe physical violence during pregnancy.

### Impact of intimate partner violence on antenatal care visits

Results from sensitivity analyses suggested that India’s records and discarded Bangladesh records did not introduce significant bias in analyzing the final selected samples (Tables S2-S3 in the **Online Supplementary Document**). In the logistic regression analysis adjusted for covariates and survey weights, women’s frequency of ANC visits was significantly associated with exposure to LSPV during pregnancy. The associations applied not only to whether women received any ANC but also to the frequency of visits.

Exposure to LSPV was associated with an increased likelihood of pregnant women seeking ANC visits at least once. The odds of any ANC visit were more than two times higher for those who were exposed to LSPV during pregnancy (OR = 2.01; 95% CI = 1.14-3.56, *P* = 0.016). On the other hand, being exposed to LSPV during pregnancy was negatively associated with women’s probability of getting more ANC visits. The odds of receiving at least four ANC visits (OR = 0.55; 95% CI = 0.40-0.76, *P* < 0.001) or eight ANC visits (OR = 0.53; 95% CI = 0.31-0.90, *P* = 0.019) were lower for respondents who had experienced LSPV during pregnancy.

At the same time, similar results were observed among women who had lifetime exposure to LSPV, which might pose more perennial and durable impacts on women’s frequency of ANC visits. For women who have had lifetime exposure to LSPV, odds of receiving at least four or eight ANC visits for their latest births were respectively 0.55 (95% CI = 0.41-0.74) and 0.47 (95% CI = 0.29-0.77) times lower compared to those who had never been exposed to LSPV in lives (**Table 6**).

**Table 6:** Associations between intimate partner violence and antenatal care visits for respondents who gave birth in the past 12 months (n = 4467)*

	Had any ANC visits (>0) for the latest birth		Had at least four ANC visits (≥4) for the latest birth		Had at least 8 ANC visits (≥8) for the latest birth	
**OR (95% CI)**	***P*-value**	**OR (95%CI)**	***P-*value**	**OR (95%CI)**	***P-*value**
**During pregnancy exposure to IPV**						
Exposed to PV during pregnancy						
*No*	ref		ref		ref	
*LSPV*	2.01 (1.14-3.56)	0.016	0.55 (0.40-0.76)	<0.001	0.53 (0.31-0.90)	0.019
*SPV*	2.35 (0.87-6.31)	0.091	1.11 (0.64-1.93)	0.707	0.85 (0.37-1.92)	0.691
Exposed to EV during pregnancy						
*No*	ref		ref		ref	
*Yes*	1.17 (0.62-2.33)	0.623	0.74 (0.49-1.13)	0.16	1.04 (0.63-1.72)	0.889
Exposed to SV during pregnancy						
*No*	ref		ref		ref	
*Yes*	0.48 (0.18-1.26)	0.136	1.11 (0.59-2.11)	0.749	0.46 (0.21-1.00)	0.052
**Lifetime exposure to IPV**						
Ever exposed to PV						
*No*	ref		ref		ref	
*LSPV*	1.35 (0.76-2.39),	0.304	0.55 (0.41-0.74)	<0.001	0.47 (0.29-0.77)	0.002
*SPV*	1.96 (0.82-4.68)	0.127	1.10 (0.67-1.81)	0.703	0.74 (0.36-1.50)	0.4
Ever exposed to EV						
*No*	ref		ref		ref	
*Yes*	1.57 (0.83-2.95)	0.162	0.84 (0.57-1.25)	0.397	1.26 (0.80-2.00)	0.318
Ever exposed to SV						
*No*	ref		ref		ref	
*Yes*	0.56 (0.22-1.47)	0.241	1.03 (0.57-1.87)	0.923	0.46 (0.22-0.98)	0.043

ANC – antenatal care, EV – emotional violence, IPV – intimate partner violence, LSPV – less severe physical violence, SPV – severe physical violence, SV – sexual violence, OR – odds ratio; CI – confidence interval, ref – reference category

*Logistic regression adjusted by survey weights was used to evaluate the impact of intimate partner violence on antenatal care visits among respondents who had given births in the past 12 mo, using demographic and health surveys data from India (2019-21), Afghanistan (2015), Maldives (2016-17), Nepal (2016), and Pakistan (2017-18). Data from Bangladesh were not included in the final sample since EV and SV data were not available in the Bangladesh demographic and health surveys data (2007). All models were adjusted for covariates at the individual (personal information and marital and reproductive information) and family (household) levels. Personal information included variables are age, area of residence, education level, and working status; marital and reproductive information included variables are age at first marriage, age at first birth, parity, and marital duration; family (household) included variables are wealth index, decision power in household, relationship to household head, husband’s education level, husband’s working status, and husband’s desire for child.

## DISCUSSION

The results of this study revealed that LSPV can negatively affect ANC utilization, with effects comparable with or even worse than those of SPV. The positive association between LSPV during pregnancy and the likelihood of having any ANC visit might be an exception. This is probably because respondents were taken to ANC late in their pregnancy after experiencing PV for bruises or injuries check but not for ANC purposes. Discussions on LSPV have been relatively scarce in previous literature. Our current results on LSPV contrast with earlier claims that PV of greater severity would have a stronger impact on affecting women’s emotional and physical health [22]. We suspect that LSPV, especially during pregnancy, can lead to similar emotional and physical impairments as SPV does. Previous studies suggested that IPV is rooted in individuals’ attempts to maintain control of their partners [11]. Similar to SPV, LSPV is also closely correlated with controlling behaviors [31]. These can include acts to constrain free mobility or access to friends and relatives, which might impair women’s ability to seek health care [32]. Our findings highlighted that the negative impacts of LSPV are not negligible. On the one hand, exposure to LSPV itself can negatively affect ANC utilization. On the other hand, previous evidence shows that IPV tends to be repetitive, with an exacerbation in frequency and severity over time [33]. Many severe injuries caused by SPV had a previous presentation with overlooked less severe injuries [34,35].

Though our analysis did not yield a significant relationship between exposure to SPV and ANC utilization, its negativities still exist. The insignificance might be attributed to the relatively small sample size of women who had experienced SPV. Moreover, previous studies proposed that women exposed to SPV might have a lower chance of being pregnant or suffering from miscarriages [36]. Thus, this group of women might be excluded in this analysis.

Another point we aimed to stress was that exposure to IPV during pregnancy and throughout life can both negatively affect women’s ANC utilization. One explanation is that lifetime exposure to IPV is highly correlated with IPV exposure during pregnancy, as our analysis revealed that most IPV happens both during pregnancy and throughout respondents’ lives. Another explanation might be the controlling nature of IPV. Even if women were not directly exposed to PV during pregnancy, they could persistently experience accompanying controlling behaviors, which impair their incentives and limit their sources to seek ANC help during pregnancy [37].

With this study we highlighted the importance of identifying potential victims of IPV during pregnancy, particularly those who have been exposed to LSPV, at health care facilities. Unlike many SPV behaviors that might violate the law in nature, LSPV is more prevalent but difficult to supervise under the legislative system. Though all South Asian countries in our study have implemented laws combating domestic violence, the enforcement remains challenging [38,39]. Therefore, the health system has a unique opportunity to identify victims and initiate interventions. Previous literature has documented potential obstacles for health care providers in identifying and intervening in IPV, including a lack of time and training, reluctance to initiate IPV conversations, uncaring attitudes, stigma, and victims’ financial dependencies [40-42]. To address these concerns, we suggest implementing supportive policies and interventions, such as developing screening methods and training modules for health care providers.

Previous studies have suggested similar patterns of IPV impacts in other countries. These countries include but are not limited to Sub-Saharan Africa, Ghana, Bangladesh, Malawi, India, Iran, and Pakistan [1,3,18,19,22-24]. Consistent patterns were also detected in many lower and middle-income countries, including Southeastern Asia, Sub-Saharan Africa, and East Africa [43,44]. In this case, we hope that our emphasis on the intolerance of any severity of PV during either lifetime or pregnancy exposure could be generalized to these regions and other lower and middle-income countries facing prevalent IPV and high maternal and neonatal mortality rates broadly.

### Study limitations

We exclusively accessed the impacts of exposure to IPV during pregnancy on women’s frequency of ANC visits in South Asia. This was less discussed in previous studies, and we assume it is due to insufficient direct data on during-pregnancy IPV experiences. We hope this could help assess the long-term impacts of lifetime exposure to IPV on ANC visits. Additionally, we differentiated between the severities of PV and suggested that the harms of LSPV were not emphasized enough before. Eventually, most existing studies on IPV and ANC utilization were localized. We hope this study on the entire region could provide a broader picture in understanding the IPV’s impacts on greater populations. Some limitations of this study are described below.

First, respondents might have difficulty remembering their past experiences and thus introduce recall bias. Second, respondents may lie or be reluctant to talk about their experiences even if they were violated, leading to underreported IPV prevalence. Due to the sensitive nature of IPV questions, misclassification could introduce unmeasurable risks and errors in the data collection process. In return, results were biased toward the null as some associations between ANC utilization and violence might not be captured. It is possible that IPV has a stronger impact on ANC utilization. Third, since this is a cross-sectional study, we cannot infer causality between experiences of IPV and ANC utilization. On the other hand, cross-sectional studies did not allow assessments of changes in ANC utilization over time. A more comprehensive understanding of IPV’s impacts on ANC utilization may be obtained by longitudinal studies. Fourth, in this study, we approximated IPV exposure during pregnancy by analyzing IPV exposure in respondents who had been pregnant within the same past-12-month period. Additionally, the study may not capture the experiences of women who have never been pregnant, limiting the generalisability of the findings to all women in South Asia. Fifth, this study did not include other potential negative outcomes such as maternal morbidity and mortality. Eventually, though we controlled some confounding variables, other uncontrolled confounders such as women’s access to transportation could have influenced the results. Further research is needed to understand the broader impact of IPV on maternal health in South Asia.

## CONCLUSIONS

The negativities of LSPV on inadequate ANC visits among pregnant women were relatively overlooked. Health providers and local communities need to quickly discern women at risk in South Asia, establish secure and trustworthy relationships with victims, and develop protective interventions to better promote the well-being of women and children in this region.

## Additional material


Online Supplementary Document
