# Supplementary material for: Overlooked impact of less severe physical violence on antenatal care visits: Findings from South Asia
Source: J Glob Health. 2023 Nov 17;13:04155. doi: 10.7189/jogh.13.04155 (PMC10654549; doi:10.7189/jogh.13.04155)
Supplement: Online Supplementary Document [file jogh-13-04155-s001.pdf]

ONLINE SUPPLEMENTARY DOCUMENT

Title: Overlooked Impact of Less Severe Physical Violence on Antenatal Care Visits: Findings from South Asia

Authors: Ling Liu1* (http://orcid.org/0009-0004-0781-8718), Di Liang1* (http://orcid.org/0000-0003-2569-1605), Saeed Anwar2 (http://orcid.org/0000-0003-3263-4182), Zunaira Michael2 (http://orcid.org/0009-0003-0546-199X), Shrinkhala Barun Shrestha3 (http://orcid.org/0000-0002-3447-7830), Nasrin Sultana4 (http://orcid.org/0000-0001-9078-3157), Jiayan Huang1 (http://orcid.org/0000-0003-4166-6119)

*Joint first authorship.

[1]School of Public Health, Global Health Institute, Fudan University, Shanghai, China

[2]Prime Institute of Public Health, Peshawar Medical College , Peshawar, Pakistan

[3]School of Medical Sciences, Kathmandu University, Dhulikhel, Nepal

[4]Institute of Health Economics, University of Dhaka, Dhaka, Bangladesh

Table S1. IPV status of respondents who had given births in the past 12 months by country

IPV status of respondents	Number of respondents exposed to form of IPV, No. (%)				
Exposure to IPV during pregnancy	Women who had given births in the past 12 months (n = 4467)				
	India (n = 3503)	Afghanistan (n = 189)	Maldives (n = 131)	Nepal (n = 332)	Pakistan (n = 312)
LSPV	518 (14.79)	52 (27.51)	5 (3.82)	24 (7.23)	47 (15.06)
SPV	150 (4.28)	22 (11.64)	1 (0.76)	14 (4.22)	11 (3.53)
EV	343 (9.79)	64 (33.86)	9 (6.87)	27 (8.13)	73 (23.4)
SV	131 (3.74)	16 (8.47)	0 (0)	25 (7.53)	13 (4.17)

IPV – intimate partner violence, PV – physical violence, LSPV – less severe physical violence, SPV – severe physical violence, EV – emotional violence, SV – sexual violence, ANC – antenatal care.

Table S2. Sensitivity analyses including data from Bangladesh: associations between PV and ANC visits among respondents who had given births in the past 12 months (n = 4919)*

Variable	Had any ANC visits (>0) for latest birth aOR (95%CI), P	Had at least four ANC visits (> = 4) for latest birth aOR (95%CI), P	Had at least eight ANC visits (> = 8) for latest birth aOR (95%CI), P
**During pregnancy exposure to IPV**			
Exposed to PV during pregnancy			
No	Reference category	Reference category	Reference category
LSPV	1.64 (1.06 - 2.54), 0.027	0.52 (0.38 - 0.70), <0.001	0.50 (0.30 - 0.82), 0.006
SPV	1.30 (0.72 - 2.33), 0.385	1.01 (0.62 - 1.62), 0.981	0.75 (0.32 - 1.73), 0.499
**Lifetime exposure to IPV**			
Ever exposed to PV			
No	Reference category	Reference category	Reference category
LSPV	1.05 (0.73 - 1.53), 0.787	0.50 (0.38 - 0.64), <0.001	0.45 (0.29 - 0.70), <0.001
SPV	0.97 (0.57 - 1.64), 0.908	0.92 (0.61 - 1.39), 0.682	0.69 (0.34 - 1.41), 0.311

PV – physical violence, LSPV – less severe physical violence, SPV – severe physical violence, ANC – antenatal care, aOR – adjusted odd ratios, P – P-value

*Logistic regression adjusted by survey weights was used to evaluate the impact of PV exposure during different timeframes on ANC visits among respondents who had given births in the past 12 months, using DHS data from Bangladesh (2007), India (2019-21), Afghanistan (2015), Maldives (2016-17), Nepal (2016), and Pakistan (2017-18). As the Bangladesh DHS data (2007) did not include EV and SV measurements, it was not possible to assess the impacts of EV and SV on ANC visits in the sensitivity analyses presented here. All models were adjusted for covariates at the individual (personal Information and Marital & Reproductive Information) and family (Household) levels. Personal Information included variables are age, area of residence, education level, and working status; Marital & Reproductive Information Included variables are age at first marriage, age at first birth, parity, and marital duration; Family (Household) included variables are wealth index, decision power in household, relationship to household head, husband's education level, husband's working status, and husband's desire for child.

Table S3. Sensitivity analyses excluding data from India and Bangladesh: associations between PV and ANC visits among respondents who had given births in the past 12 months (n = 938)*

Variable	Had any ANC visits (>0) for latest birth aOR (95%CI), P	Had at least four ANC visits (> = 4) for latest birth aOR (95%CI), P	Had at least eight ANC visits (> = 8) for latest birth aOR (95%CI), P
**Respondents Who were Pregnant in the Past 12 Months**			
**During Pregnancy Exposure to IPV**			
Experienced PV During Pregnancy			
No	Reference category	Reference category	Reference category
LSPV	0.58 (0.23 - 1.47), 0.248	0.41 (0.21 - 0.81), 0.101	0.20 (0.06 - 0.70), 0.011
SPV	0.28 (0.06 - 1.33), 0.109	1.42 (0.47 - 4.30), 0.53	0.36 (0.05 - 2.63), 0.312
Experienced EV During Pregnancy			
No	Reference category	Reference category	Reference category
Yes	1.54 (0.57 - 4.13), 0.395	0.98 (0.47 - 2.03), 0.954	2.06 (0.80 - 5.33), 0.135
Experienced SV During Pregnancy			
No	Reference category	Reference category	Reference category
Yes	0.72 (0.22 - 2.33), 0.589	0.68 (0.27 - 1.74), 0.423	1.09 (0.25 - 4.66), 0.909
Lifetime Exposure to IPV			
Ever experienced PV			
No	Reference category	Reference category	Reference category
LSPV	0.88 (0.54 - 1.45), 0.625	0.69 (0.48 - 1.01), 0.058	0.47 (0.29 - 0.75), 0.002
SPV	0.90 (0.47 - 1.74), 0.757	1.12 (0.68 - 1.85), 0.645	0.90 (0.39 - 2.05), 0.792
Ever experienced EV			
No	Reference category	Reference category	Reference category
Yes	1.02 (0.62 - 1.69), 0.931	0.86 (0.61 - 1.20), 0.363	1.75 (1.18 - 2.60), 0.005
Ever experienced SV			
No	Reference category	Reference category	Reference category
Yes	0.98 (0.45 - 2.14), 0.958	0.63 (0.38 - 1.03), 0.066	0.76 (0.33 - 1.74), 0.52

IPV – intimate partner violence, LSPV – less severe physical violence, SPV – severe physical violence, EV – emotional violence, SV – sexual violence, ANC – antenatal care, aOR – adjusted odd ratios, P – P-value

*Logistic regression adjusted by survey weights was used to evaluate the impact of IPV on ANC visits among respondents who had given births in the past 12 months, using DHS data from Afghanistan (2015), Maldives (2016-17), Nepal (2016), and Pakistan (2017-18). Data from Bangladesh and India were not included in the final sample since EV and SV data were not available in the Bangladesh DHS data (2007), and the India DHS data (2019-21) encompassed the COVID-19 period. All models were adjusted for covariates at the individual (personal Information and Marital & Reproductive Information) and family (Household) levels. Personal Information included variables are age, area of residence, education level, and working status; Marital & Reproductive Information Included variables are age at first marriage, age at first birth, parity, and marital duration; Family (Household) included variables are wealth index, decision power in household, relationship to household head, husband's education level, husband's working status, and husband's desire for child.
